# New Insights into the Skull of *Istiodactylus latidens* (Ornithocheiroidea, Pterodactyloidea)

**DOI:** 10.1371/journal.pone.0033170

**Published:** 2012-03-21

**Authors:** Mark P. Witton

**Affiliations:** School of Earth and Environmental Sciences, University of Portsmouth, Portsmouth, United Kingdom; Raymond M. Alf Museum of Paleontology, United States of America

## Abstract

The skull of the Cretaceous pterosaur *Istiodactylus latidens*, a historically important species best known for its broad muzzle of interlocking, lancet-shaped teeth, is almost completely known from the broken remains of several individuals, but the length of its jaws remains elusive. Estimates of *I. latidens* jaw length have been exclusively based on the incomplete skull of NHMUK R3877 and, perhaps erroneously, reconstructed by assuming continuation of its broken skull pieces as preserved *in situ*. Here, an overlooked jaw fragment of NHMUK R3877 is redescribed and used to revise the skull reconstruction of *I. latidens*. The new reconstruction suggests a much shorter skull than previously supposed, along with a relatively tall orbital region and proportionally slender maxilla, a feature documented in the early 20^th^ century but ignored by all skull reconstructions of this species. These features indicate that the skull of *I. latidens* is particularly distinctive amongst istiodactylids and suggests greater disparity between *I. latidens* and *I. sinensis* than previously appreciated. A cladistic analysis of istiodactylid pterosaurs incorporating new predicted *I. latidens* skull metrics suggests Istiodactylidae is constrained to five species (*Liaoxipterus brachyognathus*, *Lonchengpterus zhoai*, *Nurhachius ignaciobritoi*, *Istiodactylus latidens* and *Istiodactylus sinensis*) defined by their distinctive dentition, but excludes the putative istiodactylids *Haopterus gracilis* and *Hongshanopterus lacustris*. *Istiodactylus latidens*, *I. sinensis* and *Li. brachyognathus* form an unresolved clade of derived istiodactylids, and the similarity of comparable remains of *I. sinensis* and *Li. brachyognathus* suggest further work into their taxonomy and classification is required. The new skull model of *I. latidens* agrees with the scavenging habits proposed for these pterosaurs, with much of their cranial anatomy converging on that of habitually scavenging birds.

## Introduction

The istiodactylid pterosaurs, a group of ornithocheiroid pterodactyloids (*sensu* [1]) best known for their unusual ‘cookie cutter’ dentition and broad, rounded snouts, were represented for over a century by a single species, *Ornithodesmus* ( = *Istiodactylus*) *latidens* Seeley, 1901 [2] from Lower Cretaceous Wealden deposits of Southern Britain [3]. The history of this species is typically convoluted for a pterosaur found in British soil during the late 1800s: the holotype (Natural History Museum, London specimen NHMUK R176) was once placed in a genus now known to represent a small theropod dinosaur, *Ornithodesmus* [4]; its initial naming is problematic and risked classification as a *nomen nudum*, and some mystery surrounds when certain *I. latidens* specimens came to light, what they were, and who saw them [3]. The taxonomy and inventory of *I. latidens* is now much clearer, with specimens accessioned across the southern UK in the Natural History Museum, London; University Museum of Zoology, Cambridge and the Museum of Isle of Wight Geology (Dinosaur Isle), Sandown. The cranial morphology once only known from *I. latidens* is now shared across a distinct pterosaur group, the Istiodactylidae, which may include up to seven taxa from Barremian – Aptian deposits of Europe and Asia: *I. latidens*; a second *Istiodactylus* species, *I. sinensis* Andres and Ji, 2006 [5]; *Haopterus gracilis* Wang and Lü, 2001 [6]; *Liaoxipterus brachyognathus* Dong and Lü, 2005 [7]; *Nurhachius ignaciobritoi* Wang *et al.*, 2005 [8]; *Longchengpterus zhoai* Wang *et al*., 2006 [9] and *Hongshanopterus lacustris* Wang *et al*., 2009 [10]. The relationships of these forms are not clear, with differing interpretations of their relationships presented in recent cladistic analyses (e.g. [11]-[13]). A fragmentary fossil from the Upper Cretaceous of Vancouver was recently suggested to record a very late record of an istiodactylid (*Gwawinapterus beardi* Arbour and Currie, 2011 [14]), but the tooth replacement pattern in this animal (replacement teeth erupting directly over existing teeth) does not match that of pterosaurs (replacement teeth erupting behind existing teeth). This rather fundamental distinction questions the pterosaurian nature of *Gwawinapterus*, and may indicate that istiodactylids remain a group exclusively known from the Lower Cretaceous.

This most complete and best preserved specimen of *I. latidens* is NHMUK R3877, an incomplete skeleton from the Aptian Vectis Formation (Isle of Wight, UK) that was monographed in detail by Reginald Walter Hooley [15] and, more recently, reviewed by Howse *et al*. [3]. NHMUK R3877 represented one of the only three-dimensionally preserved pterosaurs known for much of the 20^th^ century and it remains the only istiodactylid individual known from substantial remains that are not significantly crushed. Thus, whereas some istiodactylid species are known from more complete individuals (e.g. [5], [7]-[9], [12]), many details of istiodactylid anatomy are represented by NHMUK R3877 alone.

The skull and mandibular material of NHMUK R3877 are its most interesting features, thanks to its lancet-shaped dentition (sometimes referred to as ‘razor-edged’, see [3], [16]) and broad, rounded rostrum. The skull of this individual is incompletely known, however. The middle length of the jaws, occipital aspect and much of the mandible have broken away, leading most authors to conclude that the NHMUK R3877 skull is primarily represented by two, non-articulating pieces (e.g. [3], [15], [17]-[18]). The posterior component reveals a relatively tall orbital region, preserving most of a reclined, partially closed and slender orbit; the posterior region of a large nasoantorbital fenestra that, unusually, extends beyond the jaw joint and a fragment of the articulated posterior mandible. The anterior piece, which also includes the complete mandibular symphysis, contains the anterior end of the nasoantorbital fenestra, the entire dental series and a particularly low, crestless rostrum. The two pieces are separated by sizeable lengths of maxillae and premaxillae in the upper jaw and similar lengths of mandibular rami, but the exact lengths of these missing portions are unknown. No other *I. latidens* fossils have provided a complete jaw for comparison, although another set of jaws is known: UMZC T706/R392 (accessioned in University Museum of Zoology, Cambridge, and possibly representing the lost jaws of the *I. latidens* holotype [3]). This material is badly crushed and the posterior skull remains – including the jaw joint or other hallmarks of the braincase region – are missing (MPW, pers. obs. 2007). Although more of the maxilla and mandible length are preserved in this specimen than BMNH R3877, the distortion of the skull renders it of questionable use in reconstructing the skull proportions of *I. latidens* and, at best, it only gives a minimum length of the jaws.

**Figure 1:**
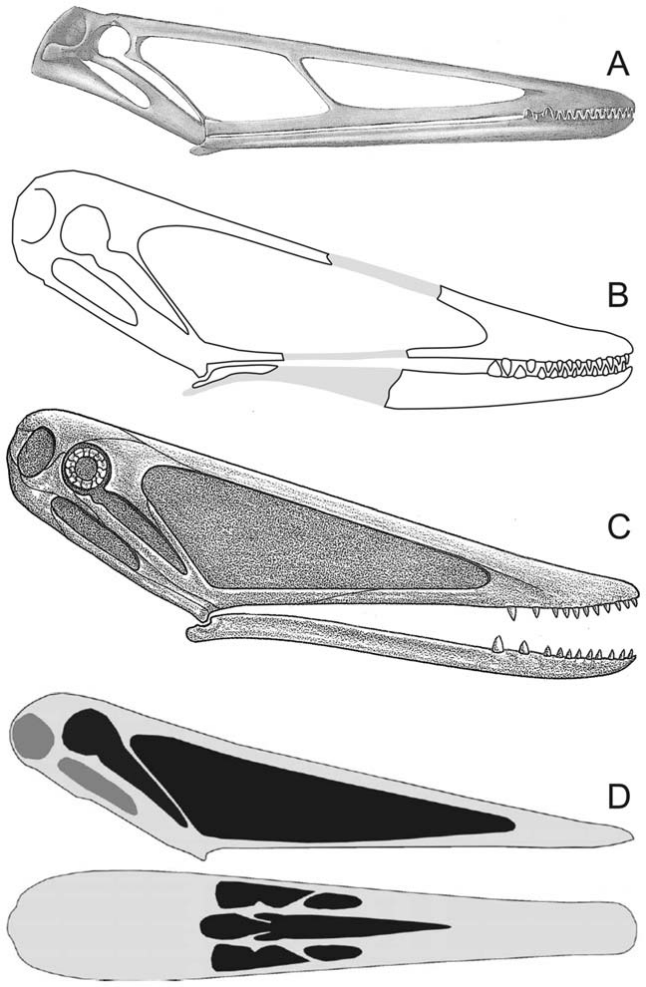
Skull reconstructions of *Istiodactylus latidens* based on NHMUK R3877. Reconstructions of this skull have changed somewhat over time, though each diagram was suggested to represent a skull 560 mm in total length. (A) Hooley [15] (B) Arthaber [17] (C) Wellnhofer [18] (D) Fastnacht [19]. All drawings modified from sources except (B) which has been redrawn and somewhat simplified.

To date, the only attempt to estimate the distance between the preserved skull pieces of NHMUK R3877 was performed by Hooley [15], who used the positioning and angles of the skull and limb bones as preserved *in situ* to estimate the size of the missing skull region. The skull pieces of NHMUK R3877 were preserved in two separate pieces of a gutter cast with their long axes oriented roughly parallel to a collection of limb bones from the same animal. Hooley assumed that the skull was continuous across both pieces and, by predicting how much material was missing from the limb bones, he deduced that approximately 300 mm of jaw had been lost. This gave a jaw length estimate of 423 mm and, via the rather low, slender-jawed reconstruction he provided in his monograph (Figure 1A), he pronounced the total skull length as 560 mm. The proportions of his reconstruction have continued to be cited by pterosaur workers for almost a century (e.g. [5], [17]-[19]) although, interestingly, other reconstructions of the *I. latidens* skull have depicted the skull as significantly shorter (Figure 1) despite citing the same overall length.

**Figure 2:**
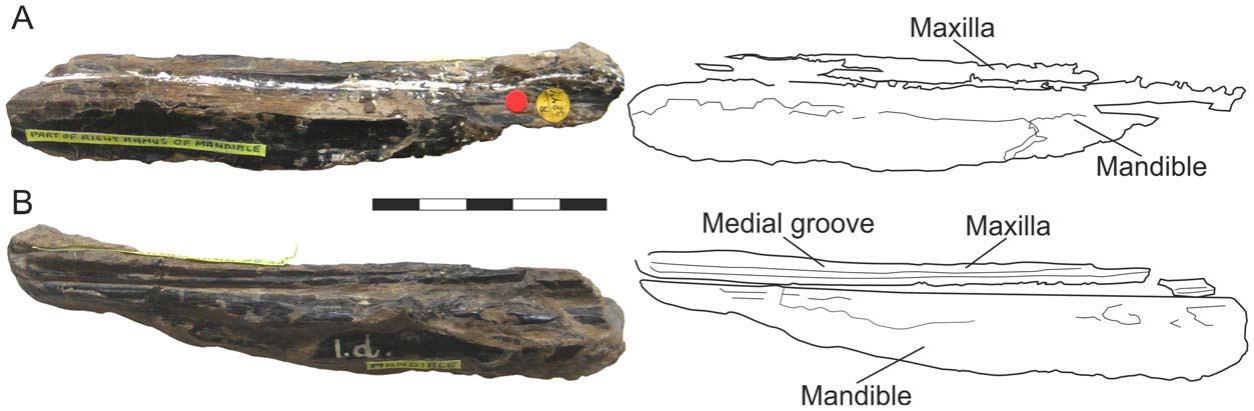
Right maxillary bar and tomial portion of right dentary of NHMUK R3977 (*Istiodactylus latidens*), the ‘missing’ jaw pieces. (A) lateral view (B) medial view. Scale bar represents 50 mm.

There is, however, a third piece of the NHMUK R3877 skull and mandible, representing short lengths of the right maxilla and mandibular ramus (Figure 2). These were documented and figured by Hooley [15] but, critically, were not considered in his reconstruction. Hooley did not even take the distinctively thin maxillary morphology of this piece into account for his restoration of the *I. latidens* skull (Figure 1A), a confusing occurrence given that he describes the ‘thin, strap-like maxilla’ in the same publication [15]. Following Hooley, this piece has not been considered in any subsequent work on this specimen. A reappraisal of this overlooked portion of NHMUK R3877 suggests it represents almost the entire missing portion of maxilla and some of the associated mandible, leaving perhaps only millimetres of jaw length missing. This allows for a minimal, and perhaps more accurate, jaw length estimate for this specimen. It suggests that the skull is much shorter skull than postulated by Hooley and that *I. latidens* one of the most distinctive and derived istiodactylids known to date. This new information is used to evaluate the phylogenetic relationships of *Istiodactylus latidens*, and has some bearing on the suggestion that *I. latidens* was a pterosaurian scavenger.

## Methods

A small analysis of 10 pterodactyloid taxa and 74 discrete characters, coded in Mesquite ([20]; version 2.75, available from http://mesquiteproject.org) and analysed in TNT ([21], program and documentation available from the authors and at www.zmuc.dk/public/phylogeny) was used to assess the relationships of *I. latidens* to other istiodactylids. *Pterodactylus*, *Coloborhynchus* and *Pteranodon* were used as outgroup taxa. Novel characters were generated for some aspects of the cranium and mandible, but postcranial characters were primarily taken from the pterosaur character list of Lü *et al*. [13]. Characters offering no resolution to pterodactyloid or ornithocheiroid relationships were omitted from this list, reducing the 55 postcranial characters of Lü *et al*. [13] to 37. Because many limb bones of *I. latidens* are missing parts of their diaphysis [15] and some doubt now exists over the methods Hooley used to reconstruct the lengths of missing bones in NHMUK R3877, many characters using limb bone metrics were not scored for *I. latidens* despite their use in previous systematic analyses of this species [5]. Characters were scored using descriptions and diagrams in appropriate literature (File S1; Table S1) except for *I. latidens* and *Pterodactylus*, which were scored from specimens (note that no permit was necessary to study the NHUMUK specimens). *Lonchengpterus zhoai* and *Nurhachius ignaciobritoi* were considered distinct taxa here despite recent suggestions that they are synonymous [12]. These taxa do not code identically in this analysis and, moreover, their synonymy was not supported in the phylogenetic analysis of the publication that synonymised them [12]! The TNT analysis used here was run using the ‘New Technology Search’ option with ‘Sectional search’ and ‘Tree fusing’ checked (default settings). Multiple-state characters were treated as unordered. Characters that exhibited multiple states for were treated as polymorphic, and bootstrap values were calculated using the ‘standard’ search and 10,000 replicates. Six MPTs were recovered with a consistency index of 0.846 and retention index of 0.72.

### Systematic palaeontology

Pterosauria Kaup, 1834 [22]

Pterodactyloidea Plieninger, 1901 [23]

Ornithocheiroidea Seeley, 1891 ([24] *sensu* [1])

Istiodactylidae Howse *et al*. 2001 [3]

Istiodactylus Howse *et al*. 2001 [3]

### Istiodactylus latidens

Seeley, 1901 [2]

### Revised diagnosis

Istiodactylid pterosaur with teeth confined to pre-nasoantorbital portion of the rostrum; no more than 48 teeth; upper toothrow occupying less than 25 percent of jaw length; sagittal ridge on rostrum; maxillae less than half the depth of the posterodorsal extension of premaxilla, width of lacrimal process of jugal less than 15 percent of the posterodorsal extension of premaxilla; quadratojugal region of skull narrower than the posterodorsal extension of premaxilla in lateral view; jaw length no more than 2.6 times the height of the skull; skull width across quadrates three times that of jaw length.

### Material

NHMUK R3877 comprises a partial skeleton of *I. latidens* including most of the skull, a fragmentary mandible, elements of the cervical and dorsal vertebrae and several broken limb elements, mainly of the right wing (humerus, radius, ulna, carpals, pteroid, wing metacarpal and two proximal phalanges). A full inventory of NHMUK R3877 is provided by both Hooley [15] and Howse *et al*. [3]. The piece under scrutiny in this paper, comprising a slender bar of right maxilla and a tomial portion of right dentary, was figured by Hooley ([15], Plate XXXVIII, Figure 4) and associated with a (misidentified) maxillonasal bar. The ‘maxillonasal bar’ is no longer associated with the maxilla and dentary fragments but may still be found in the NHMUK collections. Hooley identified both the maxilla and dentary in this piece but was unable to, or otherwise did not, link them with the larger skull remains in either his skull length estimates or skull reconstruction. The separation of the ‘maxillonasal bar’ from these jaw fragments, presumably postdating Hooley’s illustration, may indicate that additional preparation of the specimen has taken place since Hooley’s work (one referee noted its absence from Arthaber’s *I. latidens* skull 1919 reconstruction [Figure 1B], suggesting it may have been removed shortly after the publication of Hooley’s monograph) and, possibly, permit modern workers to associate the skull components in a fashion denied to Hooley. In any case, the broken ends of the maxilla, and anterior end of the dentary, are very close morphological matches to the corresponding breaks on the major skull pieces of NHMUK R3877, with the dimensions (maxillae of 6–7 mm deep the broken ends of both the larger skull elements and medial jaw pieces), size of the medial grove (see description, below), approximate fracture profiles and mandibular displacement relative to the maxilla corroborating well across all pieces. This suggests very small quantities, perhaps only millimetres, of the jaw length have been lost. The continuity of these elements was corroborated by several witnesses during a visit to NHMUK in June of 2011. Additional, independent investigation of these elements by others corroborates the continuity of these pieces (Martill, Vidovic, Davies and O’Sullivan, pers. comm. 2011).

### Description

The broken portions of right maxilla and dentary, united along their tomial margins in matrix, are positioned as if the jaw of the animal were closed in a manner consistent with the other skull remains of NHMUK R3877. They are undistorted and well-preserved, with only very slight rounding at the extremities. The precise shape of the tomial margins of both jaws cannot be seen due to unprepared matrix along their entire lengths. The maxilla measures 126 mm long and is very slender, being no more than 6–7 mm deep at any point along its length. A shallow groove extends at mid-height along its medial face and, in lateral aspect, the dorsal surface is gently concave, continuing the profile of the corresponding bones on the greater skull pieces. The posterior 11 mm of the maxilla seems somewhat displaced from the anterior portion, but matrix obscures the nature of the break between them.

137 mm of the dentary length is preserved but most of the ventral region is missing, the deepest preserved dentary portion being only 17 mm. The dentary is somewhat laterally offset from the maxilla. It is not clear if this reflects the *in vivo* condition or slight dislocation of the mandible during preservation.

## Results and Discussion

**Figure 3:**
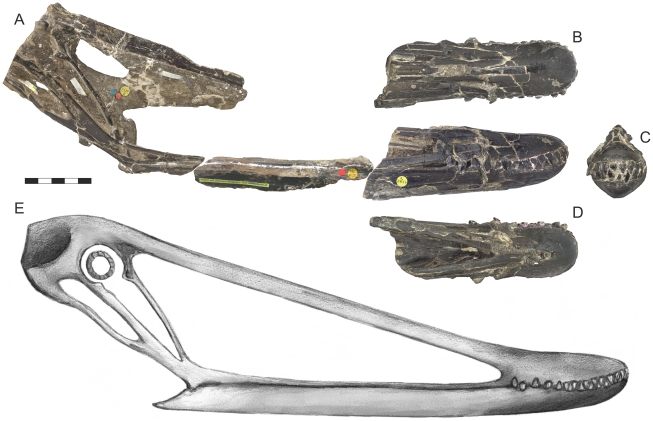
Right lateral view of the skull and mandible of NHMUK R3877 (*Istiodactylus latidens*). (A) fossil material assembled with a complete jaw length (B) new skull reconstruction. Scale bar represents 50 mm.

**Figure 4:**
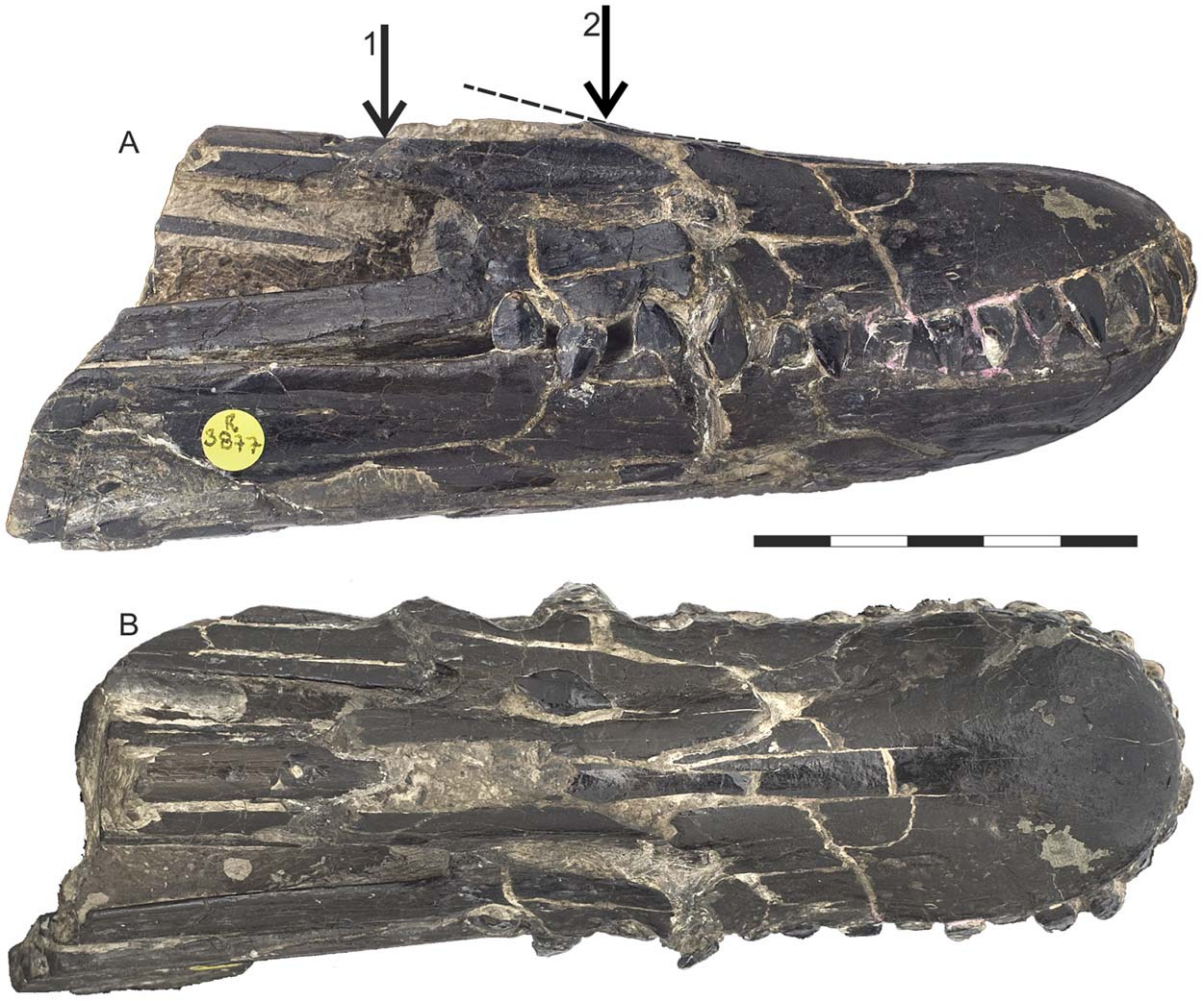
Evidence of crushing and displacement in the rostrum of NHMUK R3877. (A) Right lateral view (B) dorsal. Arrow 1 denotes displacement of the posterior region of the premaxilla from the anterior rostrum; arrow 2 indicates dorsally upturned region of the dorsal facia; dotted line shows continuation of the dorsal margin denoted by arrow 2 beyond its posterior broken surface. Scale bar represents 50 mm.

Compositing all three skull pieces together suggests a minimal jaw length of 333 mm for *I. latidens* (Figure 3), considerably shorter than the 423 mm proposed by Hooley [15]. Although the amount of missing material remains unknown, the close morphological correspondence of the broken jaw elements suggests there is little reason to assume the skull was considerably longer than this measurement. This challenges Hooley’s assumption that the skull was continuous when deposited, a challenge supported by clear indications of pre-depositional damage to the NHMUK R3877 rostrum (Figure 4). Hooley did not record any damage to the rostral portion of NHMUK R3877, but both lateral surfaces show large fractures and cracks, with the right demonstrating obvious ventral displacement of the posterior rostrum. The dorsal surface is also highly fractured with a discontinuous dorsal margin discernible in lateral view (Figure 4). This damage has displaced the entire posterior region of the rostrum, including the posterodorsal process of the premaxilla and maxillary bar. The posterior region of the toothrow is also disrupted on both lateral surfaces of the rostrum. The posterior skull piece also preserves some signs of pre- or peri-depositional damage, with the left side of the orbital region smashed and the left quadrate and various limb bones preserved within the skull cavity.

The association of matrix with these fractures indicates that such damage was not caused by diagenetic crushing or crystal growth and could not have occurred during collection. It must be assumed, therefore, that the skull was heavily damaged and disarticulated when deposited. This is consistent with the high-energy conditions predicted for deposition within a bed-scouring gutter cast. Thus, Hooley’s [15] assumption that the skull was continuous when deposited is unlikely, which casts doubt on the reliability of his skull length estimate. Note that the crushing and ventral displacement of the posterior rostrum means the angles between the two principal skull pieces cannot be used to reconstruct the skull reliably (indeed, the author failed to reproduce Hooley’s length estimate using this method). Incorporating the mid-length jaw elements into the skull reconstruction seems a far more parsimonious manner of reconstructing the skull and does not risk, unlike Hooley’s method, overestimating the skull length. It is also worth stressing that Hooley’s method of estimating the missing lengths of NHMUK R3877 cannot be tested now that the specimen has been extensively prepared.

The skull reconstruction presented here is consistent with what little UMZC T706/R392 reveals of the proportions of the *I. latidens* skull. The preserved jaw length of UMZC T706/R392 is 290 mm, including a 95.5 mm long rostrum. This provides a ratio of rostral length to preserved skull length of (0.33) and indicates that the jaws of NHMUK R3877 (with a 941 mm rostrum) must be at least 285 mm long. The minimal total jaw length of 333 mm proposed here is consistent with this.

**Figure 5:**
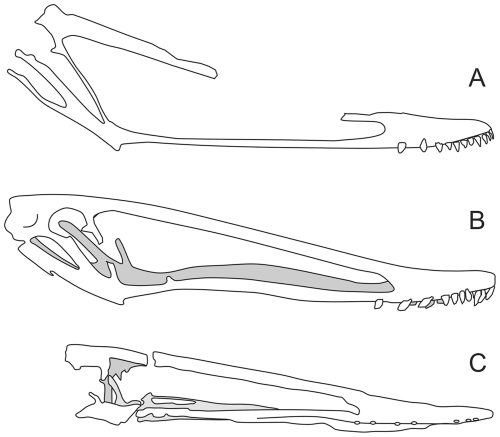
Comparisons of istiodactylid skull material, scaled to the same jaw length. (A) *Istiodactylus latidens* (NHMUK R3877) (B) *Istiodactylus sinensis* (NGMC 99-07-11) (C) *Nurhachius ignaciobritoi* (IVPP V-13288). Note the reclined, elongate orbital regions of *I. latidens* and *P. sinensis* compared to that of *N. ignaciobritoi*, and the characteristically tall, slender-boned construction of this region in *I. latidens*. (B) after Andres and Ji [5] (C) after Wang *et al*. [8].

As may be expected, this revised estimated jaw length for *I. latidens* has dramatic implications for reconstructions of its cranial morphology, resulting in a very different skull profile to that proposed by Hooley. The total preserved skull length now measures 431 mm, suggesting the entire skull was not much longer than 450 mm. This is a far cry from the estimated 560 mm skull length previously ascribed to this species and, if correct, suggests its jaws were unusually short for a pterodactyloid, occupying less than 80 percent of the preserved skull length. The upper toothrow now occupies 25 percent of the upper jaw and 27 percent of the lower, compared to less than 20 percent in Hooley’s consideration. The posterior skull is strikingly tall compared to other istiodactylids in measuring, from its tallest point to the line of the jaw, 38 percent of the jaw length. Accordingly, the posterodorsal extension of the premaxilla must have been dorsally deflected from the rostrum to connect with the posterior skull elements (as in Figure 3B), which may have been possible given the damage to the posterior rostrum of the specimen and the slight dorsal curve in the dorsal rostral margin (Figure 4). The maxilla is extremely gracile in comparison to the posterodorsal bar of the premaxilla, being almost 50 percent shallower in lateral view. Hooley [15] noted that NHMUK R3877 has a large projected skull width across the quadrates: mirroring the preserved skull along its midline suggests a width of *c.* 100 mm. Coupled with proposed jaw length estimate, this suggests *I. latidens* had a proportionally short, wide jaw with a length:width ratio of at least 0.3, a number unsurpassed in long-jawed pterodactyloids [25] and only exceeded by the short-faced pterosaur *Tapejara wellnhoferi* [26] and members of Anurognathidae (e.g. [27]). Note that the width of the jaws of NHMUK R3877 can be estimated with some confidence: the posterior skull bears none of the fracturing or crushing-induced distortion seen in the rostrum, and the rest of the NHMUK R3877 specimen is well-enough preserved that the bones articulate very well. The likelihood of the posterior skull alone being plastically distorted while the other elements are unaffected is very low. Enough of the dorsal region is preserved to reconstruct the skull apex, so a line of symmetry can be determined and the width of the skull at the quadrates estimated. Thus, it seems likely that *I. latidens* did possess a relatively short, broad skull compared to the majority of pterosaurs.

### Taxonomic implications

Assuming the reduced jaw length estimate is correct, the skull of *I. latidens* can now be seen as very distinct from other istiodactylids (Figure 5) and several autapomorphies can be added to its already sound diagnosis (see above). Most significantly, it differs much more from the skull of *Istiodactylus sinensis* (Figure 5B), an istiodactylid from the Jiufotang Formation of China ([5]) than previously appreciated. *I. latidens* has a maximum skull height of 38 percent of its jaw length, compared to 26 percent in *I. sinensis*. The toothrow of *I. latidens* is relatively short, occupying approximately 25 percent of the jaw length compared to 32 percent in *I. sinensis*, and the maxilla is much thinner in lateral profile. The lacrimal and quadrate extensions of the jugal of *I. latidens* are noticeably more slender and elongate than those of *I. sinensis* in lateral view, each being thinner than the posterodorsal extension of the premaxilla. Further cranial differences between these species have already been noted by Andres and Ji [5]: *I. latidens* has a lower tooth count (48 *vs*. 60), possesses a rostral sagittal ridge, is edentulous beneath its nasoantorbital opening and is of larger overall maximum size (2.7 vs. 4.3 m span). It should be noted that these animals remain more similar to each other than to other istiodactylids, however, through their reclined posterior skull regions, partially closed orbits and sub-orbital vacuities (Figure 5; [5], [11]). Moreover, the new skull reconstruction of *I. latidens* reveals further proportional similarities between these taxa: the lengths of their nasoantorbital fenestrae represent 83 percent of the jaw length; their rostra occupy 26–29 percent of the jaw length and their jaws comprise less than 80 percent of the overall skull length. Despite these, at least seven features (counting the slender bones of the orbital region as one character) distinguish the skulls of these animals. Postcranially, further differences have been identified: *I. sinensis* has a fused atlas axis, second wing phalanx much shorter than first phalanx and a femur more than 62 percent of the ulna length [5]. The use of these limb metrics for characterising *I. latidens* is questionable, however, as the ulna and wing phalanges of all *I. latidens* specimens known to the author are incomplete (see [15] for details), and of dubious use for taxonomic purposes.

The differences between the *Istiodactylus* species cannot be ascribed to ontogenetic influences: both NGMC 99-07-11 (the only known specimen of *I. sinensis*) and NHMUK R3877 bear bone textures and fused sutures indicative of near, or complete, osteological maturity ([28], though note that the ability to detect osteological maturity in ornithodirans has been recently questioned by some studies into dinosaurian growth and taxonomy, e.g. [29]). Nor does it seem likely that the oblique crushing affecting the *I. sinensis* skull can account for the pronounced proportional differences between these specimens. Accordingly, it must be assumed that these differences reflect taxonomic distinctions, bringing into question whether the two currently recognised *Istiodactylus* species are congeneric. Many well-established pterosaur genera are characterised by far subtler characters of their skulls than those identified between *I. latidens* and *I. sinensis* here (e.g. [30]-[32]) and a case could be made for splitting *Istiodactylus* into two, monospecific genera. The interrelationships of Istiodactylidae are not clear, however, and splitting *Istiodactylus* may unnecessarily complicate their taxonomy. Few phylogenetic analyses have included all putative istiodactylid species, but those that have included the two *Istiodactylus* species do not recover them as sister taxa [12], [33] or see them form a polytomy with another Jiufotang Formation istiodactylid, *Liaoxipterus brachyognathus* [11].

**Figure 6:**
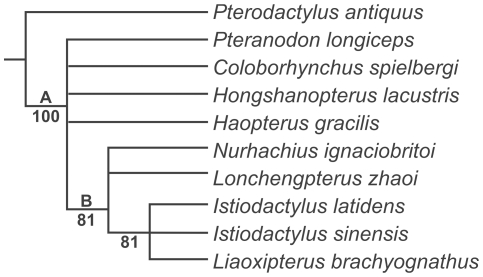
Topology of strict consensus and 50 percent majority rule consensus trees of istiodactylid interrelationships. Numbers beneath branches indicate bootstrap support. (A) Ornithocheiroidea (B) Istiodactylidae.

The small cladistic analysis of all putative istiodactylids conducted here provides little resolution on this issue (Figure 6), despite the inclusion of numerous new skull characters (File S1). The consensus tree of the four, equally parsimonious trees of istiodactylid relationships found here agrees with that of Andres and Ji [11] in finding *I. sinensis*, *I. latidens* and *L. brachyognathus* as the most derived istiodactylids currently known in an unresolved polytomy [5], [11]. Suggested synapomorphies of this clade include narrowed orbits; the presence of a suborbital vacuity; migration of the ventral subtemporal fenestra region well above the ventral orbital margin; posterior elongate posterior skull region; nasoantorbital fenestrae over 80 percent of the skull; extension of the nasoantorbital fenestra posterior to the jaw joint; a broad, rounded jaw tip in dorsal or ventral aspect; pre-narial rostrum less than 30 percent of the jaw length; an expanded jaw tip; lancet-shaped teeth with carinae; teeth restricted to the anterior 33 percent of the jaw and teeth extending beyond the posterior margin of the mandibular symphysis. This clade is supported by a good bootstrap value (83 percent) but probably lacks resolution because *L. brachyognathus* is only known from a broken mandible and four incomplete teeth, thus only providing data for nine out of the 74 characters in this analysis. *L. brachyognathus* and *I. sinensis* are identically coded in all comparable characters and only differ from *I. latidens* in one character, their larger tooth counts. It seems unlikely that such morphological subtleties can define three genera, suggesting the erection of a distinct genus for *I. sinensis* is inappropriate despite its distinction from *I. latidens*. There is, however, some question over whether *I. sinensis* would be better referred to *Liaoxipterus* (even acknowledging their limited comparability) or *Istiodactylus*. The similarities between *L. brachyognathus* and *I. sinensis* have been noted previously [12] and, given their occurrence in the same formation, it is possible that they are congeneric, or perhaps entirely synonymous. A detailed comparison between these forms, and perhaps the discovery of more substantial remains of *L. brachyognathus*, may be needed to verify these suggestions, so the existing taxonomy is provisionally retained here.

**Figure 7:**
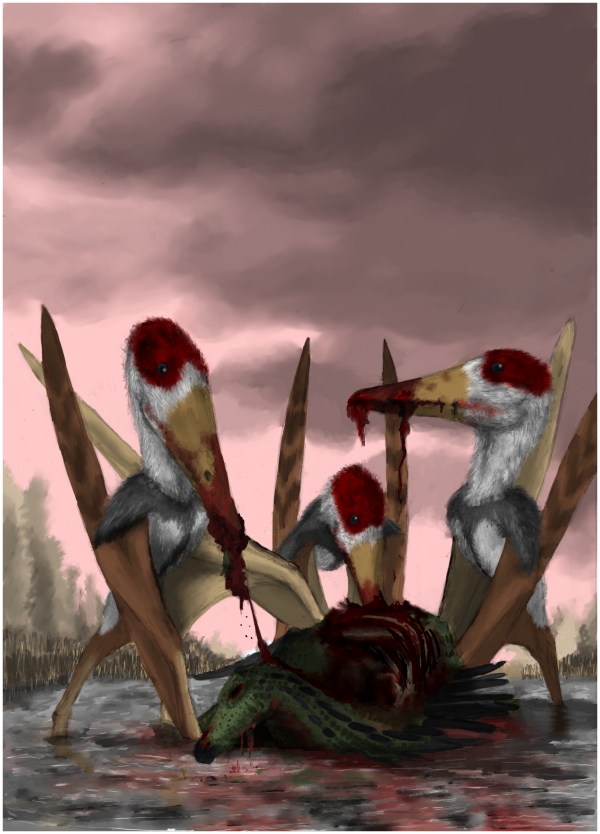
Life restoration of a group of *Istiodactylus latidens* dining on a stegosaur carcass in a shallow, Lower Cretaceous riverbed.

A second find of interest from this analysis is that *Haopterus* and *Hongshanopterus* form an unresolved polytomy with the non-istiodactylid ornithocheiroids *Coloborhynchus* and *Pteranodon*. *Haopterus* and *Hongshanopterus* have been considered istiodactylids by Lü *et al*. [12]-[13], [33] and Wang *et al*. [10], but, in this analysis, they lack synapomorphies of Istiodactylidae including a non-helical jaw joint; a nasoantorbital fenestra between 60–80 percent of jaw length; retroarticular processes over 5 percent of the jaw length; a bony odontoid at the tip of the mandible; a mandibular symphysis under 33 percent of the jaw length; less than 18 teeth in each jaw; loss of recurved dentition and the development of lancet-shaped teeth with labiolingually-compressed crown margins. Istiodactylidae is here considered to only contain five taxa (*Nurhachius*, *Lonchengpterus*, *Liaoxipterus*, *Istiodactylus* and *Istiodactylus*), with *Haopterus* and *Hongshanopterus* representing Ornithocheiroidea *incertae sedis*. *Lonchengpterus* and *Nurhachius* are found within Istiodactylidae but represent relatively basal forms compared to *Liaoxipterus* and *Istiodactylus*.

### Functionality of the Istiodactylid Skull

The features that so readily characterise the istiodactylid skull invite some discussion of their lifestyle and diet, as their unique cranial and dental morphology suggests specialised foraging habits unlike those of other pterosaurs. The diets of istiodactylids have received some previous investigation, with Fastnacht [19] performing the most detailed study of their habits to date. Using basic biomechanical calculations on the skull of *I. latidens*, Fastnacht [19] predicted a duck-like ‘hold and filter’ feeding mechanism for istiodactylids (although the possibility of more generalised feeding habits was also mentioned). His conclusions are questionable because he reconstructed the ventral profile of the *I. latidens* skull as too broad across the rostrum (compare Figure 1D with the specimen photos in Figure 3), too narrow across the quadrates and overly long in the jaw. Accordingly, his model resembles an elongated anseriform skull in ventral view and, as perpetuated by Wellnhofer [18] and Unwin [34], gives credence to the idea of istiodactylids as ‘duck-billed pterosaurs’. Such comparisons are misleading: in actuality, closed istiodactylid jaws form a circular cross-section that is nothing like the very broad, dorsoventrally compressed and often spatulate bills of anatids. The ‘razor-edged’, lancet-shaped teeth of istiodactylids are not suggestive of a filter-feeding apparatus, and a duck-like lifestyle, or appearance, does not seem likely for istiodactylids.

Most workers have interpreted istiodactylids as piscivores [6], [15], [18], [35], citing their elongate jaws and the structure of their teeth as evidence of this lifestyle. The teeth of istiodactylids argue against such a lifestyle, being quite unlike the procumbent, enlarged and recurved teeth that form ‘fish grabs’ in other toothed pterosaurs (e.g. rhamphorhynchines, ornithocheirids) that are ideally suited to spearing and holding slippery prey. By contrast, the relatively short, labiolingually-compressed, tightly-interlocking and ‘razor-edged’ teeth lining istiodactylid jaw tips seem better suited for shearing mouthfuls of food from larger sources [3] than grapping small fish. The rostra of derived istiodactylids are also proportionally short and wide, a condition contrasting with the longer, streamlined jaws of likely-piscivorous pterosaurs [36]. The anatomy of istiodactylids contrasts so much with other likely pterosaurian piscivores that they seem unlikely to have regularly adopted piscivorous habits themselves.

Howse *et al*. [3] and Unwin [34] considered that istiodactylids were vulture-like obligate scavengers, a lifestyle indicated by their broad rostra and slicing teeth. In their brief exploration of this idea, Howse *et al*. [3] suggested that twisting and pulling motions of the head could be used to tear a chunk of meat away once gripped and sheared by the teeth. Comparisons between the skulls of modern birds that independently acquired diets almost exclusively comprised of carrion (Aegypiinae, Cathartidae and, to a lesser extent, caracaras) support this idea. The skulls of scavenging birds can be readily distinguished from those of other raptors by their mosaic of strong and weak elements [37]-[39] and, in comparing the metrics of scavenging bird skulls with other raptors, Hertel [39] documented a number of features linked to a scavenging lifestyle, primarily linked to this mosaic of mechanically strong and weak skull elements. In particular, Hertel noted that scavenger maxillae, rostra and mandibles are relatively weak because, without having to subdue live prey, they can exert almost total control over the forces incurred on their skull during feeding. By contrast, their occipital faces are unusually broad, their rostra particularly hooked and their mandibular rami atypically deep. The broad occipital aspect has been interpreted because a need for increased neck musculature to pull, twist and rend the head during feeding, an efficient means to tear flesh and resist dorsoventral bending. Deepening of the mandible at the expense of lateral expansion correlates with observations that scavenging birds often pull morsels of food directly back from cadavers, perhaps because of the often crowded nature of their foraging habits [39]. Their orbits are also relatively small as, unlike predatory birds, they do not have to search for animals attempting to remain undetected nor carefully judge attacks on prey items [39]. Support for this stems from the well-documented correlation between eye size and visual acuity [40] and that scavenging birds bear reduced binocular vision and foveas than predatory raptors [41]. These characters make scavenging birds some of the most readily identifiable ecomorphs of all raptorial birds and potentially very identifiable in the fossil record [39]. The convergent development of scavenging features in several avian clades suggests that analogous features may be identifiable in animals of similar general bauplans, like pterosaurs. As explored below, istiodactylid skulls – and particularly that of *I. latidens* – appear to show a similar blend of carnivorous adaptations without any reinforcement for predation, suggesting that the scavenging hypothesis is the most compelling habit for these pterosaurs suggested to date (Figure 7).

Both Howse *et al*. [3] and Ősi [16] have noted that istiodactylid skulls are well-equipped for carnivorous habits. Their ‘razor-edged’ teeth are well-suited to shearing flesh and their unusually broad muzzles allow for sizeable portions of meat to be procured with each bite. These adaptations suggest that istiodactylids may have regularly fed on large prey items that had to be reduced into smaller pieces before swallowing. There is some reason to suspect that the jaw muscles of istiodactylids were large, too: the retroarticular processes of *I. sinensis*, *Lonchengpterus* and *Nurhachius* are much longer than those of most pterodactyloids, with the suspension-feeding ctenochasmatid *Pterodaustro guinazui* being the only species with retroarticular process of comparable size [42]. In modern crocodilians, such processes are indicative of relatively large pterygoideus musculature and the development of strong bites [43]. The development of a strong bite is of obvious utility to animals regularly required to remove chunks of meat from prey. The elongate, wide occipital face of *I. latidens* enlarges the potential area for neck musculature anchorage which, like modern vultures, may serve to assist in pulling and rending morsels of food from carcasses. The relatively great depth of the *I. latidens* skull may reflect increased resistance against dorsoventral bending compared to other, typically lower-skulled, pterosaurs, another useful adaptation to pulling flesh away from cadavers.

By contrast, other aspects of the istiodactylid skull suggest little mechanical strength. Their maxillae are slender compared to their jaw lengths and particularly so in *I. latidens*, which are more slender for their length than those of any other pterosaur. Their rostra and mandibular symphyses are also shallow, and bones around the orbital region of both *Istiodactylus* species are, to greater and lesser extents, relatively slender compared to other ornithocheiroids. These features may indicate that, like modern vultures, istiodactylids were able to control the forces sustained throughout their skulls during feeding and did not risk straining relatively weak components by subduing lively active prey. Further indication for a ‘controlled’ feeding strategy comes from their short toothrows, which indicate prey was reliably gripped in a proportionally small region of their jaws. The lack of macropredatory adaptations such as fang-like teeth or hooked talons correlates with this hypothesis: istiodactylid skulls seem well suited to eating large prey items but ill-equipped to immobilise large animals themselves. *Istiodactylus latidens* demonstrates the greatest development of possible features linked to scavenging habits, suggesting it was perhaps the pterosaur most adapted to this lifestyle. Finite element analysis of istiodactylid skulls may shed further light on these observations.

Several other features of istiodactylid functional anatomy are relevant to this hypothesis. The orbits of *Istiodactylus* are proportionally small compared to those of presumed predatory pterosaurs (such as the closely related ornithocheirids): if pterosaur orbit size correlates with some aspects of visual acuity as it does in modern raptors (see above), this may indicate a reduced need to find hidden prey. Istiodactylids were probably powerful fliers, as evidenced by distally warped deltopectoral crests and deep sterna that enlarge the area for downstroke musculature attachment (see [15], Plate XXXIX, Fig 2, Plate XL, figs. 3, 4; also [44]). The importance of flight to scavenging birds cannot be overstated: their success as obligate scavengers is strongly linked to their ability to find, travel to, and consume carcasses before terrestrial carnivores [45]. The detailed flight performance of istiodactylids remains uninvestigated, but the wing ecomorphology of *Nurhachius* has been compared to modern soaring birds in principle component analysis and may have been suited to the low-energy soaring required to search for and reach carrion [46]. There is also some indication that istiodactylids were better suited for terrestrial launches, like modern vultures, than the aquatic launches that other members of Ornithocheiroidea seem well adapted to [47]. While istiodactylids possess the ‘warped’ deltopectoral crests linked to aquatic launching in some pterosaurs [47], they are not elongated along the diaphysis, suggesting their forelimb adductor musculature was not as large as those of water-launching ornithocheiroids. In addition, the istiodactylid scapulocoracoid is somewhat more gracile than those of ornithocheiroids, perhaps corroborating the idea of relatively slight flight musculature in istiodactylids compared to their close relatives. The sedimentological context of the istiodactylid fossil record agrees with their scavenging in terrestrial settings by being strongly skewed towards freshwater deposits (e.g. [12,48v49]), or brackish sediments with strong terrestrial input [3]. There therefore seems a rich ground for enquiry into the possibility of scavenging habits in istiodactylids and, in addition to further reappraisal of NHMUK R3877, this may prove a worthwhile avenue of future research.

## Supporting Information

File S1 **Character list for phylogenetic analysis.**
(DOCX)

Table S1 **Data matrix for phylogenetic analysis.**
(XLSX)
