# Supplementary material for: New Insights into the Skull of Istiodactylus latidens (Ornithocheiroidea, Pterodactyloidea)
Source: PLoS One. 2012 Mar 21;7(3):e33170. doi: 10.1371/journal.pone.0033170 (PMC3310040; doi:10.1371/journal.pone.0033170)
Supplement: File S1 — Character list for phylogenetic analysis. (DOCX) [file pone.0033170.s001.docx]

Supplementary material 1: character list for phylogenetic analysis

Characters

1. Sagittal headcrest along middle of skull absent (0), present (1) (Andres and Ji 2008).
2. Supraoccipital headcrest absent (0), present (1) (Unwin 2003).
3. Premaxillary headcrest absent (0), present (1) (Bennett 1994).
4. Occipital face postoventrally facing (0), subhorizontal (1) (Unwin 2003)
5. Orbit open and/or pear shaped (0), at least one and a half times higher

than wide (1) (modified from Andres and Ji 2008).

1. Suborbital vacuity absent (0), present (1) (Andres and Ji 2008).
2. Subtemporal fenestra open (0), narrowed (1) (New character).
3. Subtemporal fenestra positioned behind (0) or beneath (1) orbit (New character).
4. Lacrimal process projecting into orbit absent (0), present (1) (Andres and Ji 2008).
5. Quadrate and posterior jugal processes robust (0) or slender (1) in lateral profile (modified from Andres and Ji 2008).
6. Helical jaw joint absent 0) or present (1) (Andres and Ji 2008).
7. Skull length posterior to jaw joint occupies less than 20 per cent of skull length (0), more than 20 per cent (1) (modified from Unwin 2003).
8. Skull height less than 30 per cent of jaw length (0), or more than 30 per cent (1) (New character).
9. Nasoantorbital fenestra less than 60 per cent of skull length (0), 60 – 80 per cent of skull length (1), more than 80 per cent (2) (modified from Kellner 2003).
10. Posteriormost region of the nasoantorbital fenestra anterior to jaw joint (0), posterior (1) (Lü et al. 2008a).
11. Maxilla subequal in height with posterodorsal extension of premaxilla (0), or considerably lower (1) (New character).
12. Jaw width less than 25 per cent of jaw length (0), more (1) (New character).
13. Pre-narial rostrum more than 30 per cent of jaw length (0), less (1) (New character).
14. Rostral index over 3 (0), less than 3 (1) (Martill and Naish 2006).
15. Broadly ‘U’-shaped jaw tips in dorsoventral profile absent (0), present (1) (New character).
16. Palatal ridge absent (0) or present (1) (Bennett 1994).
17. Anterior rostral profile gently tapering (0) or convex (1) in lateral view (modified from Andres and Ji 2008).
18. Laterally expanded jaw tips absent (0), present (1) (modified from Kellner 2003).
19. Jawline straight (0), upturned (1) (modified from Kellner 2003)
20. Retroarticular process short (0), or over 5 per cent of mandible length (1) (New character).
21. Mandibular crest absent (0), present (1) (Kellner 2003, Unwin 2003).
22. Bony ‘odontoid’ at tip of mandibular symphysis absent (0), present (1) (New character).
23. Mandibular symphysis less than 33 per cent of jaw length (0), more (1) (modified from Kellner 2003, Unwin 2003).
24. Majority of tooth spacing more than adjacent tooth width (0), less (1) (modified from Andres and Ji 2008).
25. Tooth number over 18 in each jaw (0), between 12 – 18 (1), less than 12 (1).
26. Teeth present (0), or absent (1) (Bennett 1994).
27. Teeth under nasoantorbital fenestra (0), or not (1) (modified from Andres and Ji 2008).
28. Tooth size markedly different along toothrow (0), uniform (1) (modified from Kellner 2003).
29. Teeth with labiolingually compressed ‘razor’ margins absent (0), present (1) (modified from Lü et al. 2008a).
30. Recurved dentition present (0), absent (1) (modified from Andres and Ji 2008).
31. Toothrow occupies more than 33 per cent of jawline (0), or less (1) (modified from Andres and Ji 2008).
32. Over 75 per cent of mandibular toothrow occurring within mandibular symphysis (0), or extending well beyond it (1) (New character).
33. Postexapophyses on cervical vertebrae absent (0); present (1) (modified from Howse 1986).
34. Lateral pneumatic foramen on centrum of the cervical absent (0); present (1) (Lü et al. 2010).
35. Mid-series cervicals short (0); elongate (1); very elongate (2) (Bennett 1994).
36. Cervical ribs present (0); highly reduced or absent (1) (Unwin 2003).
37. Neural arch of cervicals high (0); depressed down onto, or even confluent with, the centrum (1) (modified from Lü et al. 2008a).
38. Neural spines of mid-series cervicals tall, spike-like (0); low or absent (1) (modified from Lü et al. 2010).
39. Notarium absent (0); present (1) (Bennett 1994).
40. Sternum rectangular (0); triangular (1); semicircular (2) (modified from Lü et al. 2010).
41. Cristospine of sternum unconstricted (0); constricted (1) (modified from Lü et al. 2010).
42. Coracoid at least two thirds up to similar length to scapula (0); longer than scapula (1) (Unwin 2003; Kellner 2003).
43. Coracoid with well-developed brachial flange absent (0); present (1) (Kellner 2003).
44. Coracoidal contact surface with sternum articulation surface flattened, lacking posterior expansion (0); articulation surface oval, with posterior expansion (1) (Kellner 2003).
45. Proximal surface of scapula elongated (0); sub-oval (1) (modified from Lü et al. 2010).
46. Shape of scapula elongate (0); stout with constricted shaft (1) (Unwin 2003; Kellner 2003).
47. Appendicular bones with thin cortex and wide lumen absent (0); present (1) (Unwin 2003).
48. Forelimb less than 3.2 times length of hind limb (0); over 3.2 times length of hind limb (1) (Unwin 2003).
49. Pneumatic opening in palmar surface of humerus absent (0); present (1) (Unwin 2003; Kellner 2003).
50. Pneumatic opening in anconal surface of humerus absent (0); present (1) (Unwin 2003; Kellner 2003).
51. Deltopectoral crest of humerus antero-posteriorly elongate with rectangular shape (0); warped (1) (modified from Lü et al. 2010).
52. Distal end of humerus D-shaped (0); triangular (1) (modified from Bennett 1994).
53. Diameter of radius more than half the ulna (0); less (1) (Bennett 1994).
54. Ulna considerably shorter than dorsals+sacrals (0); similar in length to dorsals+sacrals (1) (modified from Lü et al. 2010).
55. Ulna less than 150 per cent of humerus (0), over 150% (1) (modified from Lü et al. 2010).
56. Ulna/tibia ratio less than 0.9 (0), over 0.9 (1) (modified from Lü et al. 2010).
57. Ornithocheiroid carpus absent (0); present (1) (modified from Lü et al. 2010).
58. Pteroid less than 30% length humerus (0); 30-60% (1) (Unwin 2003; Kellner 2003).
59. Metacarpals I-III disparate lengths (0); the same length (1) (modified from Lü et al. 2010).
60. Contact between distal syncarpal and metacarpals I-IV all four in contact (0); only I and IV contact syncarpal (1); only IV contacts the syncarpal (2) (modified from Lü et al. 2010).
61. Pes of similar size to manus (0), or considerably smaller (1) (New character).
62. Manus digit iv (wing-finger) less than 60 per cent of wing length (0); or over 60 per cent (1) (modified from Lü et al. 2010).
63. Manus digit iv (wing-finger) phalange 1 compared to length of tibiotarsus shorter to 1.5 times longer (0); 1.5-2.0 times longer (1); more than twice the length (2) (Kellner 2003).
64. Anterior profile, in lateral view, of pubis convex or straight (0); slightly concave (1) (modified from Lü et al. 2010).
65. Pubis and ischium fused to form a plate with a straight ventral margin that meets the posterodorsal margin at an acute angle (0); with convex ventral border, ischium that projects below level of the pubis and obtuse posterior apex (1) (modified from Lü et al. 2010).
66. Prepubis transversely expanded (0); conjoined prepubes forming H shape (1) (modified from Lü et al. 2010).
67. Femur caput directed inward at about 135° (0); directed steeply almost parallel to long axis of femur shaft (1) (Unwin 2003).
68. Fibula less than 80 percent the length of the tibia (0); reduced to a small splint or lost altogether (1) (modified from Lü et al. 2010).
69. Length of metatarsal III compared to tibia <30% (0); >30% (1) (Kellner 2003).

Taxa list and data source

*Pterodactylus antiquus* (data source: Wellnhofer 1975, pers. obs.)

*Coloborhynchus spielbergi* (Veldmeijer 2003)

*Pteranodon longiceps* (Bennett 2001)

*Haopterus gracilis* (Wang and Lü 2001)

*Hongshanopterus lacustris* (Wang *et al*. 2009)

*Liaoxipterus brachyognathus* (Dong and Lü 2005)

*Nurhachius ignaciobritoi* (Wang *et al*. 2005; Lü et al. 2008b)

*Istiodactylus* *sinensis* (Andres and Ji 2006)

*Istiodactylus latidens* (pers. obs)

References

Andres, B. and Ji, Q. 2006. A new species of *Istiodactylus* (Pterosauria, Pterodactyloidea) from the Lower Cretaceous of Liaoning, China. Journal of Vertebrate Paleontology, **26**, 70-78.

Andres, B. and Ji, Q. 2008. A new pterosaur from the Liaoning Province of China, the phylogeny of the Pterodactyloidea, and the convergence in their cervical vertebrae. Palaeontology, **51**, 453-469.

Bennett, S. C. 1994. Taxonomy and systematics of the Late Cretaceous pterosaur *Pteranodon* (Pterosauria, Pterodactyloidea). Occasional Papers of the Natural History Museum, University of Kansas, **169**, 1-70.

Bennett, S. C. 2001. The osteology and functional morphology of the Late Cretaceous pterosaur *Pteranodon*. Palaeontographica Abteilung A, **260**, 1-153.

Dong, Z. and Lü, J. 2005. A new ctenochasmatid pterosaur from the Early Cretaceous of Liaoning Province. Acta Geologica Sinica, **79**, 164-167.

Kellner, A. W. A. 2003. Pterosaur phylogeny and comments on the evolutionary history of the group. *In*: Buffetaut, E. and Mazin, J. M. (eds.) Evolution and Palaeobiology of Pterosaurs, Geological Society Special Publication, **217**, 105-137.

Howse, S. C. B. 1986. On the cervical vertebrae of the Pterodactyloidea (Reptilia: Archosauria). Zoological Journal of the Linnean Society, London, **88**, 307-328.

Lü, J., Unwin, D. M., Xu, L., and Zhang, X. 2008a. A new azhdarchoid pterosaur from the Lower Cretaceous of China and its implications for pterosaur phylogeny and evolution. Naturwissenschaften, **95**, 891-897.

Lü, J., Xu, L. and Ji, Q. 2008*b*. Restudy of *Liaoxipterus* (Istiodactylidae, Pterosauria), with comments on the Chinese istiodactylid pterosaurs. Zitteliania, **B28**, 229-242.

Lü, J., Unwin, D. M., Jin, X., Liu, Y. and Ji, Q. 2010. Evidence for modular evolution in a long-tailed pterosaur with a pterodactyloid skull. Proceedings of the Royal Society B, **277**, 383-389.

Martill, D. M. and Naish, D. 2006. Cranial crest development in the azhdarchoid pterosaur *Tupuxuara*, with a review of the genus and tapejarid monophyly. Palaeontology, **49**, 925-941.

Unwin, D. M. 2003. On the phylogeny and evolutionary history of pterosaurs. In: Buffetaut, E. and Mazin, J. M. (eds.) Evolution and Palaeobiology of Pterosaurs, Geological Society Special Publication, **217**, 139-190.

Veldmeijer, A. J. 2003. Description of *Coloborhynchus speilbergi* sp. nov. (Pterodactyloidea) from the Albian (Lower Cretaceous) of Brazil. Scripta Geologica, **125**, 35-139.

Wang, L., Li, L., Duan, Y. and Cheng, S. L. 2006. A new iodactylid [*sic*] pterosaur from western Liaoning, China. Geological Bulletin of China, **25**, 737-740.

Wang, X. and Lü, J. 2001. Discovery of a pterodactylid pterosaur from the Yixian Formation of western Liaoning, China. Chinese Science Bulletin, **46**, 1112-1117.

Wang, X., Kellner, A. W. A., Zhou, Z. and Campos, D. A. 2005. Pterosaur diversity and faunal turnover in Cretaceous terrestrial ecosystems in China. Nature, **437**, 875-879.

Wang, X., Campos, D. A., Zhou, Z. and Kellner, A. W. A. 2008. A primitive istiodactylid pterosaur (Pterodactyloidea) from the Jiufotang Formation (Early Cretaceous), northeast China. Zootaxa, **18**, 1-18.

Wellnhofer, P. 1970. Die Pterodactyloidea (Pterosauria) der Oberjura-Plattenkalke Süddeutschlands. Bayerische Akademie der Wissenschaften, Mathematisch- Wissenschaftlichen Klasse, Abhandlugen, **141**, 1-133.
